# Effects of depressive symptoms and peripheral DAT methylation on neural reactivity to alcohol cues in alcoholism

**DOI:** 10.1038/tp.2015.141

**Published:** 2015-09-29

**Authors:** C E Wiers, E Shumay, N D Volkow, H Frieling, A Kotsiari, J Lindenmeyer, H Walter, F Bermpohl

**Affiliations:** 1 National Institute on Alcohol Abuse and Alcoholism, Laboratory of Neuroimaging, National Institutes of Health, Bethesda, MD, USA; 2 National Institute on Drug Abuse, National Institutes of Health, Bethesda, MD, USA; 3 Department of Psychiatry, Socialpsychiatry and Psychotherapy, Hannover Medical School, Hannover, Germany; 4 Salus Clinic, Lindow, Germany; 5 Department of Psychiatry and Psychotherapy, Charité Universitätsmedizin Berlin, Berlin, Germany; 6 Berlin School of Mind and Brain, Humboldt-Universität zu Berlin, Berlin, Germany

## Abstract

In alcohol-dependent (AD) patients, alcohol cues induce strong activations in brain areas associated with alcohol craving and relapse, such as the nucleus accumbens (NAc) and amygdala. However, little is known about the influence of depressive symptoms, which are common in AD patients, on the brain’s reactivity to alcohol cues. The methylation state of the dopamine transporter gene (DAT) has been associated with alcohol dependence, craving and depression, but its influence on neural alcohol cue reactivity has not been tested. Here, we compared brain reactivity to alcohol cues in 38 AD patients and 17 healthy controls (HCs) using functional magnetic resonance imaging and assessed the influence of depressive symptoms and peripheral DAT methylation in these responses. We show that alcoholics with low Beck’s Depression Inventory scores (*n*=29) had higher cue-induced reactivity in NAc and amygdala than those with mild/moderate depression scores (*n*=9), though subjective perception of craving was higher in those with mild/moderate depression scores. We corroborated a higher DAT methylation in AD patients than HCs, and showed higher DAT methylation in AD patients with mild/moderate than low depression scores. Within the AD cohort, higher methylation predicted craving and, at trend level (*P*=0.095), relapse 1 year after abstinence. Finally, we show that amygdala cue reactivity correlated with craving and DAT methylation only in AD patients with low depression scores. These findings suggest that depressive symptoms and DAT methylation are associated with alcohol craving and associated brain processes in alcohol dependence, which may have important consequences for treatment. Moreover, peripheral DAT methylation may be a clinically relevant biomarker in AD patients.

## Introduction

Alcohol dependence is a chronic relapsing disorder, characterized by continued drinking despite an awareness of negative consequences. People often start drinking because of the rewarding, hedonic effects of alcohol, which are induced by dopamine (DA) release in the nucleus accumbens (NAc).^1, 2^ With repeated exposure to alcohol, the cues associated with it (for example, the sight of a bar or beer bottle) become conditioned over the course of addiction. These cues, then, also lead to DA increases in mesolimbic reward pathways, thereby acting as a ‘motivational magnet’ and triggering alcohol consumption.^3^ Indeed, neuroimaging studies have shown that alcohol cues evoke strong responses in mesolimbic brain areas in alcohol-dependent (AD) patients, including the NAc and amygdala, which are associated with craving,^4, 5^ and relapse after abstinence.^6, 7, 8^

Besides positive incentives, negative reinforcement motives, such as the reduction of depressive symptoms or anxiety, also play a key role in alcoholism, especially in later stages of the disease.^9, 10^ Alcohol dependence and depression are highly comorbid^11, 12^ and alcoholism is often associated with dysphoria, that is, low mood,^9^ including blunted DA responses to natural rewards.^2^ DA projections from the ventral tegmental areas to the striatum have an important role in both reward and mood,^12^ and it was recently shown that AD patients exhibit impaired striatal reward anticipation, which correlated negatively with depression scores.^13, 14^ However, little is known as to whether depressive symptoms and/or positive and negative reinforcement motives of craving are associated with alcohol cue reactivity, which is the first goal of the current study. We expected that mesolimbic cue reactivity and positive reinforcement craving were especially strong in patients with low depression scores, and that patients with higher depression scores would predominantly report negative reinforcement craving.

The DA transporter 1 (DAT) gene is implicated in both substance dependence and depression and could contribute to the comorbidity between these two disorders. For example, the DAT1/SLC6A3 VNTR polymorphism modulates drug-induced cue reactivity in the striatum in AD patients,^15^ striatal DAT availability and the severity of alcohol withdrawal.^16^ As the DAT1 9 R allele has been associated with reduced DAT expression,^17^ it may be that the allele leads to accumulation of synaptic DA, which results in increased reward processing. Lower striatal DAT availability has also been reported in patients with major depressive disorder,^18, 19^ possibly in response to reductions in DA neurotransmission.

The expression of DAT is also affected by the methylation state of the DAT promoter area. Preclinical studies demonstrate that elevated DAT methylation is related to reduced expression of DAT protein in the rat cortex and striatum.^20^ Although there is currently no evidence for this relationship in humans, it has been shown that DAT methylation of peripheral blood cells is elevated in AD patients compared with controls^21^ and negatively associated with craving.^21, 22^ Therefore, the second goal of this study was to investigate the relationships between the DAT promoter methylation state of peripheral blood cells (from now on: ‘DAT methylation’), alcohol cue reactivity and its interaction with depressive symptoms. Moreover, we explored whether DAT methylation could predict alcohol craving, and relapse 1 year after abstinence.

To accomplish this, we studied 38 recently detoxified male AD patients and 17 healthy controls (HCs) on an alcohol cue reactivity task in a 3-Tesla magnetic resonance imaging scanner. Peripheral blood measures were taken to assess DAT methylation state. We aimed to replicate the initial finding that the DAT promoter methylation state in blood is elevated in AD patients compared with HCs, and that the degree of DAT methylation covaries with alcohol craving.^21, 22^ In addition, we hypothesized that if DAT methylation affects its expression and if peripheral DAT methylation is reflective of DAT methylation in the brain, then elevated peripheral DAT methylation will be associated with stronger alcohol cue-induced blood oxygenation level dependent responses in mesolimbic brain areas (that is, NAc and amygdala), and could predict alcohol relapse. Given previous findings of reduced DAT availability in depression,^18, 19^ we also expected elevated peripheral DAT methylation in AD patients with higher depression scores and greater negative reinforcement craving.

## Materials and methods

### Participants and questionnaires

The Ethical Committee of the Charité Universitätsmedizin Berlin approved the study and a total of 38 AD inpatients and 17 HCs matched for age, years of education, intelligence (WAIS matrices)^23^ and body mass index (*P*>0.09) were included. All the participants were Caucasian males. The patients were recruited from an inpatient clinic and controls were recruited via online advertisements. All the participants gave written informed consent before the study.

Exclusion criteria for all the participants were a history of neurological dysfunctions, axis I psychiatric disorders according to DSM-IV criteria other than alcohol (in the AD group) and nicotine dependence (M.I.N.I. plus, an International Neuropsychiatric Interview).^24^ None of the participants met criteria for major depressive disorder according to DSM-IV. For controls, potential participants with scores above 8 on the Alcohol Use Disorder Identification Test^25^ were excluded, as determined in a telephone interview before participation. The patients were recently detoxified (<6 months, minimum of 1 week) and had been suffering from alcohol dependence for 15.29±8.25 years (see Table 1). Smokers were abstinent from tobacco at least 1.5 h before scanning.

We assessed lifetime alcohol intake in both the groups by means of the Lifetime Drinking History (LDH),^26^ and, in the patient group only, severity of dependence with the Alcohol Dependence Scale (ADS).^27^ Both groups rated their alcohol craving on the 14-item version of the Desire for Alcohol Questionnaire (DAQ),^28^ which can be divided into three components: positive reinforcement/desire to drink (for example, ‘Drinking would be satisfying now’), negative reinforcement (for example, ‘Drinking now would make me feel less tense’) and ability to control drinking (for example, ‘If I started drinking now I would be able to stop’). Depressive symptoms were measured using the Beck’s Depression Inventory (BDI), a 21-question multiple-choice self-report inventory.^29^

### Assessment of DAT promoter methylation

Fasting ethylenediaminetetraacetic acid blood samples were taken directly after scanning sessions and were stored at −80 °C after collection. Automated isolation of genomic DNA from whole blood was carried out by the magnetic-bead-based NucleoMag Blood 200 μl Kit (Macherey Nagel, Düren, Germany). After bisulfite treatment of the DNA (EpiTect 96 Bisulfite Kit, Qiagen, Hilden, Germany), the DAT promoter (from −1037 to −701) was amplified by PCR using the following primers: 5′-TTGTAGGTTGGAATGGTTG-3′ (forward) and 5′-CCTAAAAAAACCATTTCCC-3′ (reverse, annealing temperature 56 °C, product length 336 bp). Amplification product of PCR was purified using Agencourt AMPure XP PCR Purification Kit (Beckman Coulter, Krefeld, Germany). Sequencing was performed to measure the methylation state of 12 CpG sites residing within the DAT promoter (from −1037 to −829; sequencing primer 5′-AAAAAATAAAACCCCC-3′, product length 208 bp) using the BigDye Terminator v3.1 Cycle Sequencing Kit (Applied Biosystems, Foster City, CA, USA). Sequencing reaction was purified by Agencourt CleanSEQ Dye-Terminator Removal Kit (Beckman Coulter) before analysis with the Applied Biosystems 3500xL DNA Genetic Analyzer (Applied Biosystems). The methylation rate of each CpG site was determined using the Epigenetic Sequencing Methylation Analysis software.

### Functional magnetic resonance imaging cue reactivity task, acquisition and preprocessing

A total of 80 cues (40 alcohol, 40 soft drink) were presented over 16 blocks (8 alcohol blocks, 8 soft-drink blocks). Per block, five cues were presented for 4 s each. Four ‘oddball’ blocks were added in which participants had to press a button with their right index finger when they would see an ‘oddball’, defined as being a picture of an animal. Oddball blocks were removed from the analysis and the cue reactivity contrast of interest was (alcohol blocks >soft drink blocks). Task duration was 6 min. Cue reactivity task specifics and data of a subsample of 30 AD patients have been reported earlier.^5^

Scanning was performed in a 3-Tesla whole-body magnetic resonance imaging scanner (Magnetom Trio Tim, Siemens, Germany) with a 12-channel head coil. A standard T2-weighted echo planar imaging sequence was used with the following parameters: sequential descending acquisition, 2 s repetition time, 25 ms echo time, 80° flip angle, 64 × 64 pixels in-plane resolution, 34 slices, 3 mm slice thickness, voxels were 3 × 3 × 3 mm^3^, 0.75-mm gap between slides, 192 × 192 mm^2^ field of view, and 140 images per session. Functional data analysis was performed with SPM8. Scans were spatially realigned, slice-time corrected and normalized to the standard echo planar imaging template. Smoothing was performed with an 8-mm full-width at half maximum Gaussian kernel. Participants did not move more than 3 mm or 3 degrees per block, and hence none of the participants were excluded on the basis of this criterion.

### Statistical analyses

#### Craving and depression scores

DAQ craving scores were normally distributed in both the groups, whereas BDI scores in the AD group were not (Kolmogorov–Smirnov test, *P*=0.011). The AD patient group was split into two depressive symptom subgroups according to the BDI cut-off scores proposed by Beck *et al.*:^29^ 0–9 indicating minimal depression (*n*=29; mean=3.23±2.63), 10–19 indicating mild depression and >19 indicating moderate depression (mild and moderate together: *n*=9, mean=14.94±3.98).^29^ BDI data of six HCs were missing. None of the participants had BDI scores indicative of severe depression (⩾30). DAQ craving scores in the minimal and mild/moderate depression group were also normally distributed (Kolmogorov–Smirnov test *P*>0.20).

#### DAT methylation

The DAT promoter methylation state of DNA isolated from ethylenediaminetetraacetic acid whole blood was calculated as the mean percentage of methylation of 12 CpG sites. Mean DAT methylation was normally distributed according to the Kolmogorov–Smirnov test in each diagnostic group (*P*>0.72) and in the two patient groups on the basis of depression sores (*P*>0.20). Despite a previous study that found that DAT methylation and age were positively correlated,^22^ in the current sample we did not find age and DAT methylation to be correlated within each group (*P*>0.38) or within both groups combined (*P*=0.48). Moreover, smoking packyears (that is, (cigarettes per day/20 × smoking duration)) did not correlate with methylation in either group (*P*>0.54). Therefore, we did not add age or packyears as covariates to our analyses.

#### Neural alcohol cue reactivity

Three functional magnetic resonance imaging regressors (alcohol, soft drink and oddball blocks; 20 s each) were built for every subject and were convolved with the hemodynamic response function with default temporal filtering of 128 s. On the single subject level, the contrast difference (alcohol blocks >soft drink blocks) was calculated. We used anatomically defined bilateral amygdala and ventral striatum as our regions of interest (ROIs) using the human anatomical WFU Pickatlas.^30^ These two ROIs were also used in our previous alcohol studies.^5, 31^

#### Group comparisons and correlations

Mean beta values from all voxels in each ROI were extracted using MarsBaR software for SPM8 (MARSeille Boîte À Région d’Intérêt; http://marsbar.sourceforge.net), which provided an estimate of alcohol cue reactivity in each ROI. These measures were compared between groups using independent *t*-tests (Student’s *t*-test, or Welch’s *t*-test if Levene’s test for equality of variance was *P*<0.05) and correlated (Pearson’s *r*, two-tailed) with craving scores (including exploratory correlations with craving subscores) and DAT methylation state, with a significance threshold of *P*<0.05 using SPSS 22 (IBM, Armonk, NY, USA). For variables that were not normally distributed, we used the Mann–Whitney *U*-test for group comparisons and Spearman’s rank-order rho for correlation analyses. We corrected for the number of ROIs used and report both corrected and uncorrected *P*-values. Results with *P*-values of 0.05<*P*<0.1 are reported as trends. Exploratory whole-brain analyses in AD patients versus HCs and AD patients with low versus mild/moderate depression scores were calculated within SPM8, with a threshold of *P*<0.001 uncorrected and cluster size of *k*>20.

### Relapse after abstinence

One year after abstinence, alcohol relapse rates of AD patients were acquired through postal and telephone interviews.^5^ Relapse was defined as drinking alcohol within the last 12 months for at least 3 days in a row. A single lapse shorter than 3 days that was ended by the patient without further negative consequences was still considered abstinence, which is in line with previous measures of relapse.^32, 33^ Measures of craving, neural alcohol cue reactivity and DAT methylation were compared between patients who abstained or relapsed abstainers within 1 year, using independent *t*-tests in SPSS with the thresholds described above.

## Results

### Subject characteristics and behavioral craving

#### AD patients versus HCs

Table 1 lists clinical and demographic data of AD patients and HCs including mean±s.d. and *P*-values of group statistics, showing that groups did not differ in age, education or intelligence scores. AD patients reported stronger alcohol craving (DAQ sum score) compared with HCs (Student’s *t*_52_=4.90, *P*<0.0001). This effect was owing to stronger craving on the negative reinforcement (Mann–Whitney *U*=185.5, *P*=0.013) and ability to control drinking (*U*=10.5, *P*<0.001) subscales. There were more smokers in the AD group (30/38) than in the HC group (6/17) (*χ*^2^=9.90, *P*=0.002).

#### AD patients; low depression versus mild/moderate depression scores

Clinical and demographic data for the two subgroups of AD patients on the basis of severity of depressive symptoms according to BDI are reported in Table 2. The groups did not differ in age, education, intelligence, body mass index, lifetime alcohol intake or smoking characteristics (all *P*>0.05). Patients with mild/moderate depression scores, however, had higher scores on the ADS (*t*_35_=2.20, *P*=0.035) and a longer history of dependence (*t*_36_=2.15, *P*=0.038) than patients with low BDI scores. Moreover, patients with mild/moderate depression scores reported significantly stronger total DAQ craving (*t*_35_=2.31, *P*=0.027) compared with patients with low depression scores, particularly for the negative reinforcement subscale (*t*_35_=2.22, *P*=0.033). There were no group differences for the subscale positive reinforcement or ability to control. Despite a strong comorbidity between smoking and depression,^11^ there were more smokers in the low depressed (25/29) than mild/moderate depressed (5/9) group (*χ*^2^=3.88, *P*=0.049), but no between-group differences in cigarette packyears (*P*=0.18; see Table 2).

### DAT methylation

#### AD patients versus HC

**Figure 1:**
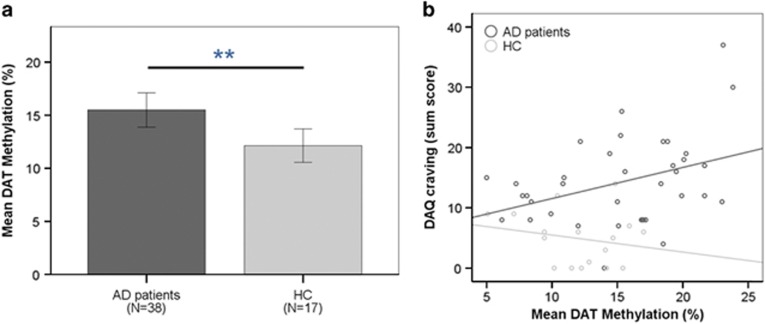
(**a**) Mean peripheral DAT promoter methylation in AD patients and HCs (mean%±s.d.). AD patients had elevated DAT methylation compared with HCs (*P*=0.008). (**b**) DAT methylation predicted DAQ craving in AD patients (*r*^2^=0.14, *P*=0.024), whereas not in HCs (*r*^2^=0.043, *P*=0.43). When comparing regression slopes between these groups, the AD group showed a stronger correlation between DAT methylation and DAQ craving than HCs (Fisher’s *z*=1.88, *P*=0.03). AD, alcohol-dependent; DAQ, Desire for Alcohol Questionnaire; DAT, dopamine transporter gene; HC, healthy control.

DAT promoter methylation state was elevated in AD patients (mean=15.30±5.14% s.d.) compared with HCs (mean=12.13±3.24% Welch’s *t*_46.7_=2.76, *P*=0.008; Figure 1a), and predicted DAQ craving in AD patients (*r*^2^=0.14, *P*=0.024), but not in HCs (*r*^2^=0.043, *P*=0.427). When comparing regression slopes between groups, the AD group showed a stronger correlation between DAT methylation and DAQ craving than HCs (Fisher’s *z*=1.88, *P*=0.03; Figure 1b). The regression between DAT methylation and craving in AD patients was particularly apparent for the negative reinforcement subscale (*r*^2^=0.172, *P*=0.011), but did not reach significance for the two other subscales (control and positive reinforcement *P*>0.22).

#### AD patients; low depression versus mild/moderate depression scores

Patients with mild/moderate depression scores had elevated levels of DAT methylation (mean=18.98±4.17%) compared with AD patients with low BDI scores (mean=14.16±4.93% *t*_36_=2.65, *P*=0.012). Although DAT methylation predicted DAQ craving in AD patients pooled together, the regression did not reach significance for the AD subgroups on the basis of BDI score (*P*>0.17).

### Neural alcohol cue reactivity

All the participants responded to all four ‘oddballs’, and hence paid attention to the cue reactivity task.

#### AD patients versus HC

There were no group differences between AD patients and HCs for alcohol cue-induced reactivity in our ROIs. Moreover, an exploratory whole-brain analysis using a liberal threshold of alpha *P*<0.001 and uncorrected *k*>20 did not show group differences. Activations in both ROIs did not correlate with craving or methylation state in either group (*P*>0.05).

#### AD patients; low depression versus mild/moderate depression scores

**Figure 2:**
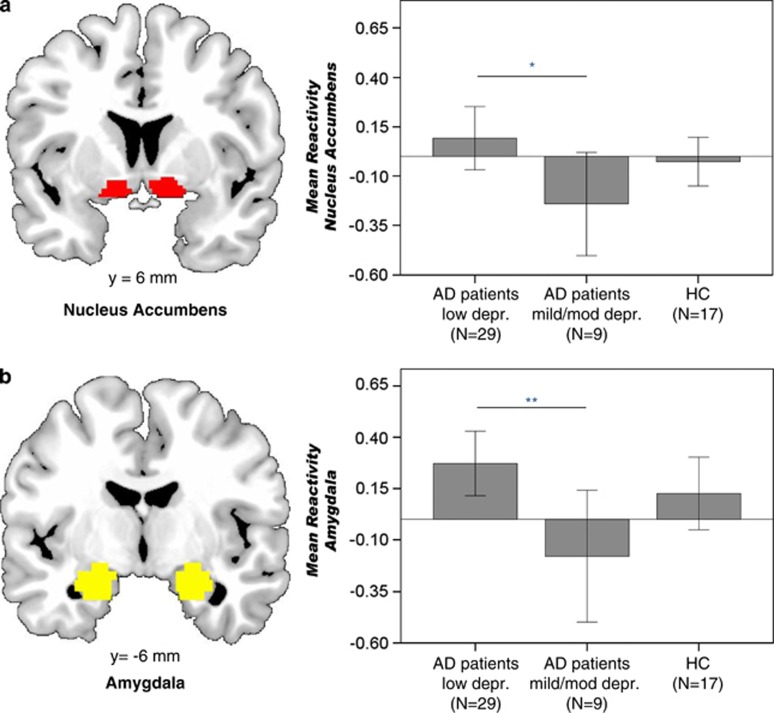
AD patients with low depression scores had stronger alcohol cue reactivity in the bilateral NAc (*P*=0.046; **a**) and bilateral amygdala (*P*=0.01; **b**) compared with AD patients with moderate/high depression scores. Regions of interest were anatomically defined. There were no group differences between AD patients (both groups pooled together, *n*=38) and HCs (*n*=17) for alcohol cue-induced reactivity. AD, alcohol-dependent; HC, healthy control; NAc, nucleus accumbens.

AD patients with low depression scores had elevated alcohol cue-induced reactivity in the NAc (*t*_36_=2.06, uncorrected *P*=0.046, corrected *P*-value for two ROIs=0.092) and amygdala (*t*_36_=2.71, uncorrected *P*=0.010, corrected *P*=.020) when compared with AD patients with mild/moderate depression (When correcting group comparisons for potential confounding factors—that is, smoking status, duration of dependence, ADS scores and DAQ craving; see Table 2; group differences in NAc (*t*=2.50, uncorrected *P*=0.018, corrected *P*=0.036) and amygdala (*t*=2.74, uncorrected *P*=0.010, corrected *P*=0.020) remained.) (Figure 2). An exploratory whole-brain analysis using a liberal threshold of alpha *P*<0.001 and uncorrected *k*>20 revealed increased alcohol cue reactivity for AD patients with low versus mild/moderate severity in medial prefrontal cortex (peak Montreal Neurological Institute [x,y,z]=[−6,44,−8], *P*<0.001 family-wise error cluster-corrected), bilateral thalamus (peak right=[6,−7,−2], *P*<0.001 uncorrected), left putamen/insula (peak=[−27,14,−8], *P*=0.027 family-wise error cluster-corrected) and right brainstem (peak=[3,−31,−38], *P*=0.038 family-wise error cluster-corrected; see Supplementary Table S1).

Only in AD patients with low depression scores, alcohol cue reactivity in the amygdala correlated positively with DAQ craving (DAQ sum: *r*=0.48, *P*=0.008) and with DAT methylation (*r*=0.42, *P*=0.022). The correlation with craving was apparent for the DAQ subscales positive reinforcement (*r*=0.37, *P*=0.049) and negative reinforcement (*r*=0.47 *P*=0.010), but not for ability to control drinking (*P*=0.90). Activation in the NAc did not correlate with craving in any group (*P*>0.13), despite a trending correlation between craving and NAc activation that we found in a subset of 30 AD patients reported earlier.^5^ Further, amygdala and NAc reactivity did not correlate with other clinical variables, including years of alcohol dependence, LDH, ADS or smoking packyears in any group (all *P*>0.1).

### Relapse prediction

**Figure 3:**
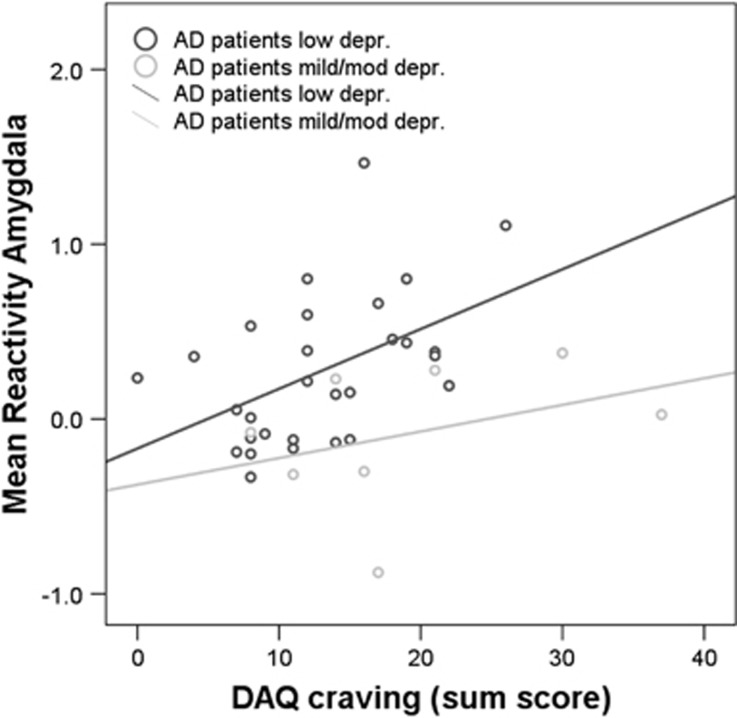
DAQ craving correlated with neural alcohol cue reactivity in the bilateral amygdala in the AD group with low depression (depr) scores (*r*=0.48, *P*=0.008), but not in the group with mild/moderate depression scores (*P*>0.1). AD, alcohol-dependent; DAQ, Desire for Alcohol Questionnaire.

A total of 29 AD patients responded by either mail or phone. Of these, 17 remained abstinent, whereas 12 relapsed within a year after abstinence. Although craving scores and alcohol cue reactivity did not differ between groups (*P*>0.05), DAT methylation tended to be higher in patients who relapsed (17.24±5.08% s.d.) than those who abstained (14.07±4.50% s.d.) at trend level (*t*_27_=1.73, *P*=0.095; see Figure 3). The two BDI groups did not differ in relapse (low depression group: 14 abstained and 10 relapsed; mild/moderate depression group: three abstained and two relapsed; *χ*^2^=0.005, *P*=0.95). However, DAT methylation predicted relapse as a trend in AD patients with low BDI scores (*t*_22_=2.07, *P*=0.051), whereas not in patients with mild/moderate depression scores (*U*=3, *P*=1; but please note that the sample size was only five in this group). Supplementary Table S2 lists the clinical and demographic characteristics of relapsers and abstainers.

## Discussion

The main finding of this study is that depression scores, as measured through the BDI, have impact on neural alcohol cue reactivity in AD patients without a comorbid diagnosis of major depression. Specifically, we found that in alcoholics, cue reactivity in the bilateral amygdala, NAc (at trend level when corrected for multiple comparisons), as well as other areas in the brain reward circuitry (for example, medial prefrontal cortex, thalamus, putamen, insula), was stronger in AD patients with low BDI scores compared with those with mild/moderate depression scores. Therefore, this indicates that AD patients with higher depression scores not only show reduced striatal reactivity to general reward processes,^13^ but also less alcohol-specific mesolimbic reward reactivity. Enhanced reactivity to drug cues in alcohol and drug abusers is thought to be a core mechanism underlying cue-induced craving, relapse and treatment success,^4, 8, 10^ which may persist even after alcohol abstinence.^3^ However, the results of our study suggest heterogeneity in mesolimbic cue reactivity and craving: we found that patients with higher depression scores failed to show increases in brain reward areas for alcohol cues, which may have consequences for treatment. For example, alcohol cue exposure therapies may be successful only in individuals with low depressive symptoms.

On a behavioral level, a reverse pattern was shown for craving ratings: patients with mild/moderate depression scores reported stronger DAQ craving than low-depressed patients, which, in line with our hypothesis, was particularly apparent for the negative, but not for the positive reinforcement, subscale. This suggests that negative reinforcement craving is predominant in patients with higher depression scores, and, indeed, it has been proposed that these patients try to reduce dysphoria with alcohol.^9, 11, 34^ Although patients with higher BDI scores also scored higher on the ADS and were generally dependent for a longer period of time which may have influenced the results, these variables were not associated with brain cue reactivity over the whole patient population, or within the subgroups. In fact, when correcting group comparisons for these potential confounding factors (that is, smoking status, duration of dependence, ADS scores and DAQ craving), the differences in cue reactivity were even stronger. Only in AD patients with low depression scores, alcohol cue reactivity in the amygdala correlated with alcohol craving (both sum, positive and negative reinforcement), whereas no associations were found in the group with higher depression scores. This further suggests that a subgroup of AD patients with higher depression scores do not show mesolimbic cue reactivity and have increased negative reinforcement craving. The association between amygdala cue reactivity and craving was previously found in a subsample 30 AD patients,^5^ although that study did not explore depressive symptoms.

Another important finding of the study is that the methylation state of the DAT promoter measured in peripheral blood cells was associated with alcoholism, craving processes and as a trend for relapse (*P*=0.095). This is consistent with an earlier finding that peripheral DAT methylation was elevated in AD versus HC^21^ and that it predicted alcohol craving.^21, 22^ Although these previous studies found a reverse correlation between DAT methylation and craving, in our study we observed a positive association, particularly for the negative reinforcement subscale. This difference may be due to different scales used: the previous studies used the Obsessive Compulsive Drinking Scale, which distinguishes the three factors obsession, interference and control impairment, but does not assess negative reinforcements.^35^ This may also explain why the subgroup with higher depression scores demonstrated higher DAT methylation. A preliminary finding from our study was that DAT methylation showed a trend for being higher in relapsing versus abstaining AD patients, as assessed 1 year after detoxification. Thus, further research on the value of DAT methylation in peripheral blood as a potential biomarker for alcoholism and its value in predicting clinical outcomes merits investigation.^36^

We did not find evidence in support of our hypothesis on a direct association between alcohol cue reactivity and DAT methylation in all the AD patients. However, after splitting the AD patients into depression groups, we found that in AD patients with low depression scores alcohol cue reactivity in the amygdala (but not the NAc) correlated with DAT methylation and craving (see above). The lack of a correlation in the group consisting of patients with higher depressive symptoms may have been owing to the small sample size.

Previous studies in AD have focused on DAT availability in the striatum^16, 37^ and involvement of the DAT1 9 R allele in striatal cue reactivity,^15^ but did not report results on the amygdala. Similarly, lower striatal DAT availability has been found in the striatum of depressed individuals.^18, 19^ Recently, however, the DAT1 9 R allele has been implicated in amygdala reactivity to salient cues.^38^ In an animal model of alcoholism, Jiao *et al.*^39^ found that chronic alcohol use increased DAT binding in the amygdala and substantia nigra in depressive-like and non-depressive-like rat strains, but only in the NAc of depressed rats. It is possible that the differences between the strains in alcohol-induced DAT binding are, at least, in part, due to genetic make-up, which, in turn, determines dopaminergic differences in reward and depression. These studies, together with our findings, corroborate the importance of the amygdala in alcohol dependence and its interaction with depressive symptoms and DAT. Although both DAT promoter methylation and the DAT VNTR polymorphism have been shown to alter DAT expression, it is not clear whether these alterations are inter-related. The DAT methylation region analyzed in our study is in the extended promoter region of the DAT1 gene, whereas the VNTR polymorphism resides in the 3′-UTR. To the best of our knowledge, it is not known whether the length of the 3′-UTR polymorphism influences the methylation profile of the promoter, which should be subject of future studies. Moreover, it remains unclear how DAT promoter methylation in the peripheral tissue relates to DAT expression and function in the brain.

Our study has some important limitations. First, AD patients were recently detoxified treatment-seeking inpatients and, therefore, the severity of depression (that is, the BDI assesses symptoms in the past 2 weeks) may have been directly associated with the severity of withdrawal symptoms that the patients were experiencing,^34^ which we did not assess. Second, we excluded AD patients with comorbid axis I psychiatric disorders (other than nicotine dependence), including depression and anxiety, which reduces the generalizability of the study to the general AD population who frequently show comorbid illnesses. However, our goal was to study the brain reward circuitry in alcohol dependence without a potential confounding factor of general anhedonia owing to a primary depressive disorder.^40^ A previous study in AD patients with comorbid depressive and anxiety disorders found that depression scores correlated with alcohol cue-induced reactivity in the precuneus and parietal lobe, but not in the mesolimbic areas in the AD patients.^41^ A likely reason for the differences between the findings of Sjoerds *et al.*^41^ and our study is their merging of patients with these comorbid diagnoses. Indeed, the scale used by Sjoerds *et al.* indicated depression scores above clinical threshold (in some of the AD population), whereas none of the patients in the current cohort had BDI scores indicating clinical levels of severe depression. A third limitation is that there were missing values in the HC group, which made it difficult to make strong conclusions about whether depression scores influenced alcohol cue reactivity in this group. Future research using a 2 × 2 factorial design with AD patients/HCs, with low/moderate depression scores may reveal whether DAT methylation is also elevated in HCs with BDI scores indicating moderate depression. Fourth, DAT methylation was measured in the peripheral blood cells and it is currently unclear whether methylation of this promoter region in blood is an accurate representation of the methylation state in brain tissue. Comparisons between epigenetic marks of DAT in peripheral tissue and the brain, either postmortem or *in vivo* using imaging techniques, are hence needed. Recent studies investigating blood and brain relationships found mixed results: some studies report strong correlations between the methylation state of brain and blood tissues,^42, 43, 44^ whereas others emphasize differences between these tissues,^45, 46^ especially in genes involved in cell differentiation.^46, 47^ Interestingly, it has been suggested that methylation in saliva rather than blood may be a better marker for brain methylation.^47, 48^ Last, our AD patients and HC group also differed in smoking behavior, which may have confounded the results. Also, the BDI cut-off scores of 10 made the group of AD patients with mild/moderate depression scores rather small and the findings, therefore, have to be replicated in larger samples.

In summary, we believe our study is the first to demonstrate reduced mesolimbic cue reactivity in alcoholic patients with higher depression scores. Moreover, peripheral DAT promoter methylation state may merit further investigation as a potential biomarker in AD patients as it (1) was greater in AD patients versus HCs, (2) was a predictor of alcohol craving in AD patients, (3) showed a trend towards predicting relapse 1 year after abstinence and (4) was associated with amygdala cue reactivity in patients with low depression scores. The study is the first ‘imaging epigenetics’ study in addiction, which, beyond traditional genetic imaging, may provide further mechanistic insight into causal pathways of gene–environment interactions and their effect on brain function.^49, 50^

## Figures and Tables

**Table 1:** Demographic and clinical characteristics of AD patients and HCs

*Characteristic*	*Alcohol-dependent patients,* N=*38*		*Healthy controls,* N=*17*		P*-value*
	*Mean*	*s.d.*	*Mean*	*s.d.*	
Age, years	44.39	7.32	42.71	9.15	0.47
Years of education	10.45	1.24	11.12	1.69	0.16^a^
WAIS, matrix reasoning	14.91^b^	4.93	17.59	5.48	0.10^a^
BMI	26.04^c^	4.56	24.38	2.26	0.16^d^
Packyears	17.86^c^	15.20	8.71	14.19	**0.018**^a^
LDH	1405.27^e^	1319.10	144.59	221.90	**0.000**^a^
Duration of dependence, years	15.29	8.25	—	—	—
Length of abstinence, days	52.63	42.77	—	—	—
Alcohol Dependence Scale	15.70^e^	7.47	—	—	—
DAQ sum	14.27^e^	7.28	4.88	4.43	**0.000**
DAQ positive reinforcement	3.24^e^	3.94	2.88	2.80	0.74
DAQ negative reinforcement	4.38^e^	3.79	1.71	1.79	**0.013**^a^
DAQ control	6.65^e^	1.87	0.29	0.59	**0.000**^a^
BDI	6.05	5.82	5.46^f^	5.07	0.93^a^

Abbreviations: AD, alcohol-dependent; BDI, Beck’s Depression Inventory Scale; BMI, body mass index; DAQ, Desire for Alcohol Questionnaire; HC, healthy control; LDH, Lifetime Drinking History; WAIS, Wechsler Adult Intelligence Scale.

a Mann–Whitney *U-*test statistics, as sample(s) are not normally distributed.

b *N*=35.

c *N*=36.

d Welch’s *t*-test was used since equality of variance was not assumed (Levene’s test *P*<0.05).

e *N*=37.

f *N*=11. *P*-values in bold are <0.05.

**Table 2:** Demographic and clinical characteristics of AD patients with low (BDI <10) and mild/moderate depression scores (BDI >10)

*Characteristic*	*Alcohol-dependent patients, low depression scores (*N=*29)*		*Alcohol-dependent patients, mild/moderate depression scores (*N=*9)*		P*-value*
	*Mean*	*s.d.*	*Mean*	*s.d.*	
Age, years	44.45	7.37	44.22	7.60	0.95^a^
Years of education	10.48	1.35	10.33	1.00	0.81^a^
WAIS, matrix reasoning	14.69^b^	4.96	15.56	5.05	0.66
BMI	25.90^c^	4.42	26.55^d^	5.31	0.73
Packyears	19.64^e^	14.89	12.53	15.76	0.18^a^
LDH	1207.72	1126.27	2121.39^d^	1768.32	0.22^a^
Duration of dependence, years	13.76	7.94	20.22	7.65	**0.038**
Length of abstinence, days	56.83	44.10	39.11	37.21	0.17^a^
Alcohol Dependence Scale	14.25^c^	6.57	20.22	8.66	**0.035**
DAQ sum	12.90	5.93	19.25^d^	9.79	**0**.**027**
DAQ positive reinforcement	2.79	3.22	4.88^d^	5.87	0.19
DAQ negative reinforcement	3.69	3.36	6.88^d^	4.42	**0.033**
DAQ control	6.41	1.99	7.50^d^	1.07	0.15^a^
BDI	3.29	2.63	14.94	3.97	**0.000**^a^

Abbreviations: AD, alcohol-dependent; BDI, Beck’s Depression Inventory Scale; BMI, body mass index; DAQ, Desire for Alcohol Questionnaire; LDH, Lifetime Drinking History; WAIS, Wechsler Adult Intelligence Scale.

a Mann–Whitney *U*-test statistics, as sample(s) are not normally distributed.

b *N*=26.

c *N*=28.

d *N*=8.

e *N*=27. *P*-values in bold are <0.05.
