# Supplementary material for: Effects of depressive symptoms and peripheral DAT methylation on neural reactivity to alcohol cues in alcoholism
Source: Transl Psychiatry. 2015 Sep 29;5(9):e648–. doi: 10.1038/tp.2015.141 (PMC5545640; doi:10.1038/tp.2015.141)
Supplement: Supplementary Information [file tp2015141x1.doc]

**Supplementary material**

Table S1. Results of a whole-brain analysis in AD patients with low > mild/moderate depression scores, with a threshold of p<.001 uncorrected, cluster size k>20. There were no activated clusters showing negative activity at this threshold. Areas in bold are thresholded p<.05 family-wise error cluster-corrected.

| **Brain region** | **Hemisphere** | **Cluster size** | **Peak Coordinates MNI (x, y, z)** | | | **t-value** | **p-value**  **FWE**  **(cluster)** |
| --- | --- | --- | --- | --- | --- | --- | --- |
| **Medial Prefrontal Cortex** | L/R | 289 | -6 | 44 | -8 | 5.12 | **.000** |
| Thalamus | L/R | 46 | 6 | -7 | -2 | 4.69 | .27 |
| **Putamen/Insula** | L | 117 | -27 | 14 | -8 | 4.56 | **.027** |
| **Brainstem/Pons** | R | 106 | 3 | -31 | -38 | 4.02 | **.038** |

Table S2. Demographic and clinical characteristics of AD patients that remained abstinent or relapsed within one year after detoxification. The significant difference between abstainers and relapsers in their length of abstinence may be simply because those patients who had been abstinent longer at the time of testing, were also followed-up earlier to remain a total time of one year after detoxification. Further, there was a trend for abstainers showing higher WASI scores, used as a proxy for general intelligence, than relapsers.

|  | **Abstaining alcohol-dependent patients (N=17)** | | **Relapsing alcohol-dependent patients (N=12)** | |  |
| --- | --- | --- | --- | --- | --- |
| **Characteristic** | **Mean** | **SD** | **Mean** | **SD** | **p-value** |
| Age, years | 46.24 | 5.75 | 42.92 | 7.70 | .20 |
| Years of education | 10.29 | 1.45 | 10.58 | 1.16 | .56 ^1^ |
| WASI, matrix reasoning | 16.63 ^a^ | 4.37 | 13.09 ^b^ | 5.41 | .12 ^1^ |
| BMI | 25.71 ^a^ | 3.49 | 24.49 | 3.42 | .37 |
| Packyears | 16.73 ^c^ | 15.00 | 22.53 | 11.52 | .29 |
| LDH | 1464.62 | 1477.18 | 1414.70 | 1376.98 | .81 ^1^ |
| Duration of dependence, years | 14.29 | 7.24 | 15.50 | 10.01 | .71 |
| Length of abstinence, days | 61.06 | 33.76 | 29.25 | 27.91 | **.002** ^1^ |
| Alcohol Dependence Scale | 15.69 ^a^ | 5.31 | 18.42 | 10.52 | .42 ^2^ |
| DAQ Sum | 13.18 | 6.19 | 14.58 ^c^ | 7.26 | .58 |
| DAQ Positive Reinforcement | 3.00 | 3.37 | 2.92 | 3.96 | .65 ^1^ |
| DAQ Negative Reinforcement | 3.88 ^c^ | 3.35 | 4.83 | 4.13 | .71 ^1^ |
| DAQ Control | 6.29 ^c^ | 2.23 | 6.83 ^c^ | 1.47 | .62 ^1^ |
| BDI | 5.15 | 4.25 | 6.50 | 6.91 | .91 ^1^ |

^a^ N=16 ^b^ N=11 ^c^ N=15. ^1^ Mann-Whitney U test statistics, since sample(s) not normally distributed. ^2^ Welch’s t-test was used since equality of variance was not assumed (Levene’s test p<.05). Abbreviations: BDI Beck’s depression inventory, BMI Body mass index, DAQ Desire for alcohol questionnaire, LDH Lifetime drinking history, WASI Wechsler adult intelligence scale.


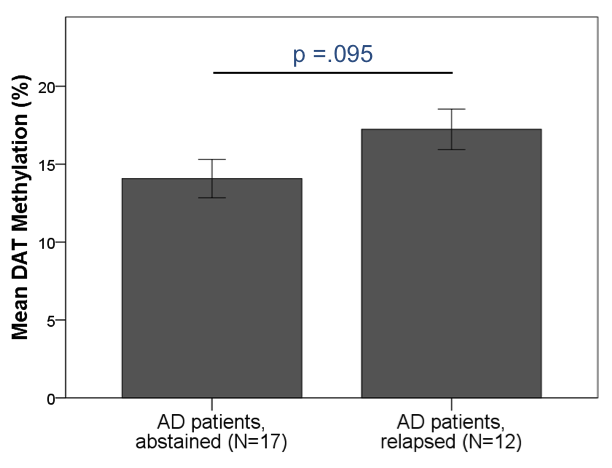


Supplementary Figure S1. Peripheral DAT promoter methylation was higher in patients who relapsed (17.24±5.08%SD) than those who abstained (14.07±4.50%SD) at trend-level (t_27_=1.73, p=.095).
